# Veratrum Viride

**Published:** 1877-04-20

**Authors:** C. J. Rademaker


### VERATRUM VIRIDE.

By C. J. RADEMAKER, M.D.

American hellebore is generally considered by the profession as a powerful vegetable poison; but having never heard of a case of fatal poisoning by this drug, and as I am in the habit of using this medicine a great deal in my practice, I concluded to make a series of experiments with it upon the lower animals, both in regard to its physiological and poisonous properties. American hellebore, when given to the lower animals, whether given in the form of a saturated tincture or the alkaloid itself in small doses, produces the following effects: First, a great increase of saliva and flow of tears, accompanied with an incessant cough, the animal at the same time dancing around the room. If now the dose is increased, either by the mouth or by hypodermic injection, vomiting sets in rapidly, the pupils become dilated, accompanied by great muscular relaxation, so that the animal is unable to walk; but at no time have I noticed tonic spasms and stiffness of the limbs, even when the drug had been given in enormous doses. Upon the circulation it has a powerful sedative effect; when given in small doses it diminishes the pulse, without producing any unpleasant symptoms. But if the medicine is now increased largely, it brings the heart down to about twenty-five beats per minute, accompanied by all the symptoms given above; but in no instance could I give a small dog enough of the alkaloid (veratria) to kill him.

One small dog I gave ten grains of veratria and then placed him under the influence of chloroform, and made a careful vivisection, so that part of the heart was exposed, which I could see and feel beating tumultuously at about twenty or twenty-five beats per minute; but after he came out of the stupor of chloroform he got up and looked about him, apparently thinking that nothing had happened; so I struck him on the head with an axe and killed him.

The stomach of this dog was taken out and found highly injected with blood over the entire mucous surface. The stomach was submitted to analysis, and three grains of veratria found; consequently five grains must have been absorbed, allowing two grains for loss, which is large.

From the above it will be seen that veratrum can not be considered a powerful poison. As for narcotic properties, it possesses none; and it can only be classed with the irritant poisons. Of course, where given in very large doses, it would produce gastro-enteritis and probably death.

Taylor, in his Treatise on Poisons, speaks of the irritant properties of black and white hellebore, but says nothing in regard to veratrum viride. In a case reported by Morgagni, (Taylor's Treatise), a half dram of the aqueous extract of black hellebore killed a man aged fifty years in eight hours. The symptoms were pain in the abdomen and vomiting. After death the whole alimentary canal was found inflamed. In one instance twenty grains of the white hellebore caused death in three hours (Taylor's Treatise). Death was preceded by vomiting of bloody mucus and cold sweats A physician prescribed medicinally one grain of veratria, divided into fifty pills, and three were to be taken at a dose (equivalent to one sixteenth of a grain), which, according to Taylor, came very near killing her. But I have not seen such results with the American hellebore on the lower animals. Medicinally I have not prescribed it in large doses, but I am satisfied that one-sixteenth of a grain of veratria will produce no poisonous effects.

THERAPEUTIC USE.

Veratrum viride is an excellent remedy in pneumonia of children, for it can be given in such a way that the child is not aware that it is taking medicine. The form in which I give it to children is the following: R Liq. potas. citratis, oz. j; tinct. veratri viridis, gtt. ii to iv or vj, according to the age of the child; a teaspoonful of the mixture to be given every two hours. This dose of course can be increased if necessary, but in a very young child I find one quarter of a drop sufficient to reduce the heart's action. In pneumonia of adults I give four to eight drops of the tincture every two hours until the pulse is reduced. I then order it to be given every four or six hours. The tincture I use is (Norwood's) made by macerating eight ounces of the rhizome to one pint of alcohol. The other treatment, such as poultices, food, and Dover's Powder for pain and rest, is not neglected, and complications of any kind. are met with proper remedies. In croupous pneumonia I always use quinine in large doses, as recommended by Juergensen. (See Ziemssen's Practice.)

In scarlatina I have found this an excellent remedy to reduce the pulse, always using it in conjunction with a bath at a temperature of 80 or 85 deg., and allowing the child to remain in it from one-half to three-quarters of an hour. This bath I use more for its effect upon the renal organs and skin than the effect it would produce upon the pulse and temperature.

During this last epidemic I treated about ninety cases of scarlet fever, out of which I lost but five. Whether this was owing to my treatment or not, I am unable to say, but will leave that to the judgment of the profession.

In dentition of children with high fever and bounding pulse, especially in those phlegmatic children that are so liable to be taken with cerebral meningitis, the veratrum, in combination with potas. bromidum, is the remedy *par excellence.*

In acute rheumatism this remedy is not less efficacious, sufficient quantity being given to reduce the pulse. In this disease it may be given in combination with opium or morphia, which ease pain, while the sedative remedy reduces the excitement. It may also be combined with the alkalies and colchicum, as in the following mixture :

**R.** Potas bicarb..................dr. iij.
Vin. colchi. sem............oz. ss.
Tinct. veratri viridis .......gtt. 60.
Aqua destil..................oz. vi.

M. ft. sol. Sig. Tablespoonful every two or three hours.

In typhoid and other low forms of fever, and in organic disease of the heart where it becomes necessary to repress the circulation, the veratrum will be found of great benefit.

In conclusion, I would say that veratria may be considered a powerful irritant poison, but at the same time death is not likely to be produced by its action upon the cerebro-spinal system or upon the heart. If death occur after its administration, it is probably the sequence of inflammation of the stomach and bowels.—*Louisville Medical News,*

